# Implementation of the International Health Regulations in Somaliland supports multisectoral response to COVID-19

**DOI:** 10.7189/jogh.10.020364

**Published:** 2020-12

**Authors:** Jussi Sane, Petri Ruutu, Saeed Soleman, Mohamed Elmi

**Affiliations:** 1 Department of Health Security, Finnish Institute for Health and Welfare, Mannerheimintie, Helsinki, Finland; 2 Ministry of Health Development, Hargeisa, Republic of Somaliland

The International Health Regulations, IHR (2005), represent an agreement between all Member States of the World Health Organization (WHO) to work together for global health security [1]. Through IHR, countries have agreed to build their capacities to detect, assess and report public health events, which are supporting Essential Public Health Functions overall [2]. WHO plays the coordinating role in IHR and, together with its partners, helps countries to build capacities. Many countries suffer from coordination challenges and a difficulty of involving comprehensively the sectors involved in preparedness and health security, especially when the topic traverses multiple ministries and departments [3].

Assessment of IHR capacities, including the State Party annual reporting process and voluntary external evaluation using the Joint External Evaluation (JEE) tools, have helped to identify vulnerabilities and priority actions [3]. Finland has been playing an active role in global forums promoting health security preparedness including the development of JEE tool and the assessment mechanism [4]. The ongoing COVID-19 pandemic has highlighted the multisectoral nature of health security and stressed the need to further strengthen preparedness through IHR implementation plans.

In 2016 a JEE of IHR capacities was conducted for the Federal Republic of Somalia, including Somaliland [5]. Experts from the Finnish Institute of Health and Welfare (THL) were present in the mission. Following the mission, the Somaliland Ministry of Health Development (MOHD) requested THL to provide technical support in the IHR implementation in Somaliland. The project was part of the Finnish -funded MIDA FINNSOM project, which in general aims to support Somalia to develop its health and education sectors [6] and involves engagement of diaspora experts. The objective was to support MOHD capacities in IHR coordination, communication and advocacy which in turn could facilitate the overall national planning process, related resource mobilization, and sustainability. The International Organization of Migration (IOM) was the lead partner on the ground.

The work was initiated in late 2017. Baseline analysis was performed by setting the technical findings in the JEE report specifically into Somaliland context, including field visits. Technical training in communicable disease surveillance and control, and awareness raising were implemented in interactive workshops, including case exercises on Rift Valley fever and brucellosis, and discussions with key officials in several key ministries and technical agencies. As of July 2020, three missions have been completed identifying challenges on the ground, and increasing IHR-awareness on different administrative levels in several sectors stressing the multisectoral approach in IHR implementation. The characteristics making multisectoral collaboration particularly important in Somaliland include a large proportion of the population being pastoralist taking care of a diversity of livestock in large herds, with close interface between humans and animals increasing the risk of zoonotic diseases, and potentially large impact on national economy [7].

Emphasizing local ownership and sustainability, the THL expert recommendations included formal establishment of a high-level IHR multisectoral steering group, drawing considerations from the Tripartite Guide to Addressing Zoonotic Diseases in Countries [8], and regular information sharing mechanisms in the operative level in order to support multisectoral risk assessment and incident management. The need of a legal framework to support multisectoral collaboration in communicable diseases was reviewed, highlighted by examples of existing legislation from neighboring countries.

The fourth THL mission was planned to specifically provide technical support in the ongoing COVID-19 response, but due to travel restrictions and other challenge, this work was performed through teleconferences. The lessons learned from the response in Somaliland so far have indicated that the work performed on IHR implementation have made Somaliland better prepared for the pandemic. Guided by the IHR training provided by THL experts, the Government of Somaliland was in a better position to swiftly institute a whole government approach in responding to COVID-19 early in 2020. A National Coordinating committee on COVID-19 was established headed by Vice President, composed of Ministers of Health, Interior, Information and guidance, Religion affairs, and Finance. The MOHD has the technical lead on the response, composed of nine committees assigned to specific activities. A National Plan was developed following a thorough needs assessment of Somaliland’s current national capacities and in alignment with WHO’s Strategic Preparedness and Response Plan guidelines [9]. The plan has been developed to cover a period of nine months from March 2020 and will be reviewed in the course of implementation depending on how COVID-19 evolves. This National Plan was developed in consideration of the status of health care system and existing infrastructure in Somaliland, and the need to build the country's resilience given the chronic nature of emergencies that the country faces.

**Figure:**
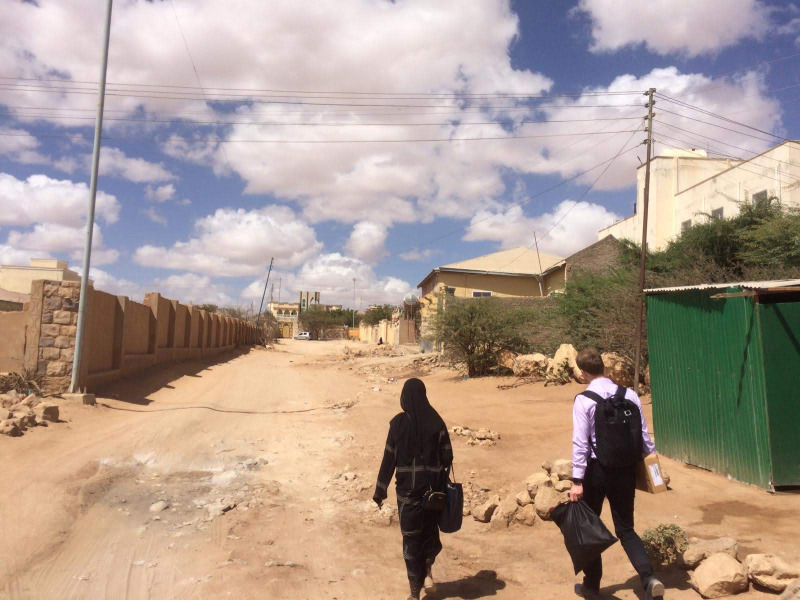
Photo: from Jussi Sane’s collection (used with permission).

However, a number of great challenges remain in the current response and in preparedness for future health emergencies. Strengthening the microbiology laboratory functions remains vital as effectiveness of any surveillance system in guiding response and decision making is dependent on sufficient laboratory capacity. Training and retaining competent public health workforce is crucial for sustainability and long-term planning. Donor support mechanisms are needed but also gradual increase in government funding over time to ensure the implementation of key priorities to mitigate effects of every-day health threats as well future pandemics.
